# Pretreatment drug resistance in a large countrywide Ethiopian HIV-1C cohort: a comparison of Sanger and high-throughput sequencing

**DOI:** 10.1038/s41598-018-25888-6

**Published:** 2018-05-15

**Authors:** Nigus Fikrie Telele, Amare Worku Kalu, Solomon Gebre-Selassie, Daniel Fekade, Samir Abdurahman, Gaetano Marrone, Ujjwal Neogi, Belete Tegbaru, Anders Sönnerborg

**Affiliations:** 1 Division of Clinical Microbiology, Department of Laboratory Medicine, Karolinska Institute, Karolinska University Hospital, Stockholm, Sweden; 2 Department of Microbiology, Immunology and Parasitology, Addis Ababa University, Addis Ababa, Ethiopia; 3 Department of Infectious Diseases, Addis Ababa University, Addis Ababa, Ethiopia; 4 Public Health Agency of Sweden, Solna, Sweden; 5 Unit of Infectious Diseases, Department of Medicine Huddinge, Karolinska Institute, Karolinska University Hospital, Stockholm, Sweden; 6 Ethiopian Public Health Institute, Addis Ababa, Ethiopia

## Abstract

Baseline plasma samples of 490 randomly selected antiretroviral therapy (ART) naïve patients from seven hospitals participating in the first nationwide Ethiopian HIV-1 cohort were analysed for surveillance drug resistance mutations (sDRM) by population based Sanger sequencing (PBSS). Also next generation sequencing (NGS) was used in a subset of 109 baseline samples of patients. Treatment outcome after 6– and 12–months was assessed by on-treatment (OT) and intention-to-treat (ITT) analyses. Transmitted drug resistance (TDR) was detected in 3.9% (18/461) of successfully sequenced samples by PBSS. However, NGS detected sDRM more often (24%; 26/109) than PBSS (6%; 7/109) (p = 0.0001) and major integrase strand transfer inhibitors (INSTI) DRMs were also found in minor viral variants from five patients. Patients with sDRM had more frequent treatment failure in both OT and ITT analyses. The high rate of TDR by NGS and the identification of preexisting INSTI DRMs in minor wild-type HIV-1 subtype C viral variants infected Ethiopian patients underscores the importance of TDR surveillance in low– and middle–income countries and shows added value of high-throughput NGS in such studies.

## Introduction

Ethiopia is heavily affected by the human immunodeficiency virus type 1 (HIV-1) epidemic with an estimated number of 700,000 infected persons, although the adult prevalence has decreased to 1.1% in 2016^1,2^. Antiretroviral therapy (ART) has been widely accessible since 2005^3^. The first-line consists of fixed-dose combinations (FDC) of two nucleoside/nucleotide reverse transcriptase inhibitors (NRTI; zidovudine (ZDV) or tenofovir (TDF) plus lamivudine (3TC) or emtricitabine (FTC)), and a non-nucleoside RTI (NNRTI; efavirenz (EFV) or nevirapine (NVP))^4^. Prevention of mother-to-child transmission (PMTCT) was launched in 2003 using single-dose nevirapine (sdNVP). In 2014, 360,000 patients were estimated to receive ART^5^. However, more than a quarter of Ethiopian patients are lost-to-follow-up (LTFU), in some regions up to 40%^4,6^. Also, the absence of monitoring of viral load and the limited availability of second-line ART, can be expected to contribute further to a high rate of therapy failure with drug resistance, as seen in other low and middle income countries (LMIC)^7,8^.

A recent report from WHO revealed a global increase in pretreatment drug resistance (PDR)^9^. In Ethiopia, there are no data so far of transmitted drug resistance (TDR) or PDR at the national level, except very limited information from some parts of the country. A study from Addis Ababa using a sensitive allele-specific polymerase chain reaction reported a 6.5% TDR prevalence^10^ and two studies from Northwest Ethiopia reported a 3.3%^11^ and a 5.6% TDR prevalence, respectively^12^. Due to this very limited information about the situation in Ethiopia with regard to HIV drug resistance, we assessed TDR by population-based Sanger sequencing (PBSS) among ART naïve patients, included in a large nationwide cohort study, and compared the results with next-generation sequencing (NGS). Thus, none of our patients were known to have been treated with ART before inclusion in the study. In addition, due to the recent introduction of integrase strand transfer inhibitors (INSTI) in certain African countries, we also analysed the NGS results for INSTI associated drug resistance mutations (DRM).

## Results

Among the 874 ART naïve HIV-1 infected patients enrolled in the study, 676 and 459 had VL data at month six and 12, respectively. Ninety (13.3%) and 61 (13.3%) had VL ≥150 copies/ml (detection limit of the assay) and 57 (8.4%) and 34 (7.4%) had VL ≥1000 copies/ml (WHO definition of virologic failure), respectively.

As described in the methodology section, baseline samples of randomly selected 490 patients (females: 58%; median age: 33 years) were tested for PDR by PBSS and assessed mutations associated with NRTI−, NNRTI−, and PI− drug classes. In addition, baseline samples of 109 virologic treatment failure (n = 71) and virologic suppressor (n = 38) patients were analyzed by NGS (females: 55%; median age: 30 years), where PDR associated with NRTI-, NNRTI-, PI- and INSTI- drug classes were considered (Table 1).

**Table 1:** Baseline sociodemographic, clinical and laboratory parameters of HIV-1 infected patients analysed with genotypic resistance testing.

Baseline parameters	PBSS	NGS
	n (%) n = 490	n (%) n = 109
**Gender**: females/males	286 (58.0)/ 204 (42.0)	60 (55.0)/49 (45.0)
**Age** (median, IQR) in years	33, 12	30, 11
**WHO clinical stage**		
Stage I	85 (17.4)	17 (15.6)
Stage II	111 (22.7)	23 (21.1)
Stage III	211 (43.1)	48 (44.0)
Stage IV	83 (16.9)	21 (19.3)
**CD4 count** (mean; SD) cells/µl	137; 93	125; 81
**HIV-1 RNA** (mean; SD) log10 copies/ml	5.27; 0.7	5.44; 0.6

PBSS: population-based Sanger sequencing; NGS: next generation sequencing.

### Outcomes of ART and sequencing

**Figure 1:**
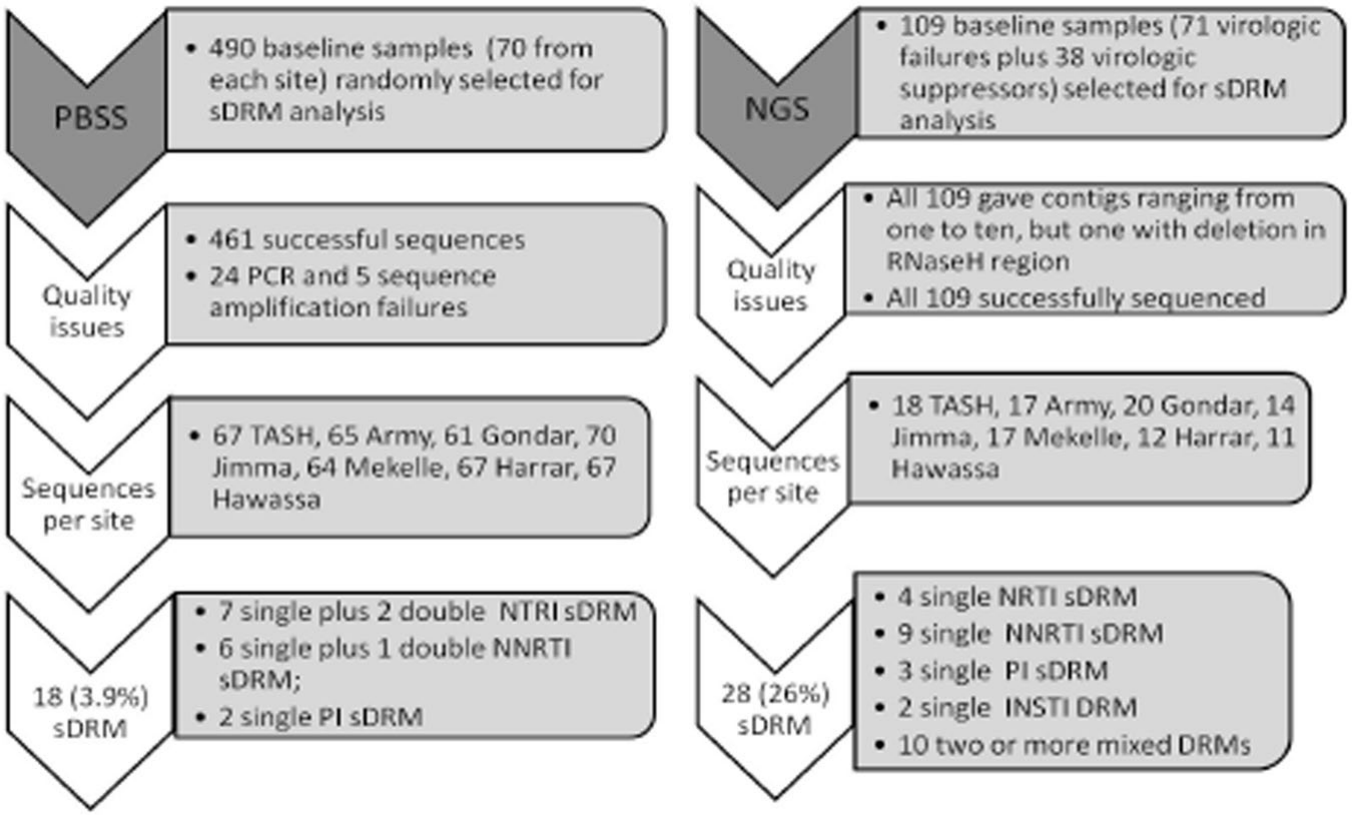
Study profile of patients. PBSS: population-based Sanger sequencing; NGS: next generation sequencing. NRTI: nucleoside analogue reverse transcriptase inhibitors; NNRTI: non-nucleoside RTI; PI: protease inhibitors; INSTI: integrase inhibitors. DRM: drug resistance mutations (surveillance drug resistance mutations were described for NRTI, NNRTI and PI).

Of the 490 patients, 408 (83.3%) were still on treatment at month six and the remaining were either LTFU (n = 33) or dead (n = 49) (Fig. 1). Plasma HIV-1 RNA (VL) was not tested in 20 subjects and among those with a VL data, 316 (81.4%) had undetectable viremia, and 72 (18.6%) detectable viremia. At month 12, 383 (78.2%) out of 490 subjects were still on treatment. VL was not tested in 114 subjects and among those with an available VL, 228 (84.8%) had undetectable viremia, and 41 (15.2%) had detectable viremia. Eleven patients had died and 14 were LTFU, respectively, between month six and 12.

For PBSS, a *pol*-sequence was obtained in 461 out of 490 (94%) samples at baseline, 47 out of 51 (93%) at month six and 30 out of 33 (91%) at month 12 (Fig. 1). For NGS, a result was obtained for all 109 samples with contigs ranging from one to ten. Of these, the best contig was selected. Three samples gave a fragmented contig, which were rectified manually. Another sample showed a large deletion in the RNaseH and integrase region. The sequences clustered with HIV-1C, except one CRF02_AG by the three subtyping tools listed in the methodology section.

### Baseline sDRM detected by PBSS and impact on treatment outcome

At baseline, 18 (3.9%) of the 461 patients with sequence data had sDRM (NRTI: n = 9; NNRTI: n = 7; PI: n = 2) (Figure 1; Table 2). None of the patients had dual drug class sDRM. Three patients had two mutations of the same drug class. There was no correlation between the presence of sDRM and study site, sex, and CD4 + T-cell count (data not shown), but the odds of having sDRM decreased significantly as participant’s age increased (OR: 0.93; 95% CI: 0.87–0.99) and increased with higher baseline viral load (OR: 2.67; 95% CI: 1.25–5.71).

**Table 2:** Drug resistance mutations detected by population based Sanger sequencing in 18 HIV-1 infected patients at baseline, months six and/or 12.

PID	First-line regimen	Baseline				Acquired DRM					
		VL*	Primary sDRM			Month 6			Month 12		
			NRTI	NNRTI	PI	VL*	NRTI	NNRTI	VL*	NRTI	NNRTI
ETH604	ZDV-3TC-NVP	4.28	—	G190S	—	5.32	—	G190S	5.29	—	—
ETH027	ZDV-3TC-NVP	4.90	—	Y181C	—	3.70	T69N, M184V	V108I, Y181C	2.36	VL <1000 cp/ml	VL <1000 cp/ml
ETH042	ZDV-3TC-NVP	5.02	—	Y181C	—	4.55	K70KR, M184V	A98G, Y181C G190A	LTFU	LTFU	LTFU
ETH646	TDF-3TC-EFV	6.93	—	Y181C, Y188C	—	6.38	K65R, K70EK, M184IV	V90I, K103N, Y181C, G190S, F227L	6.80	No sample	No sample
ETH038	TDF-3TC-NVP	6.05	T215S	—	—	5.15	Y115FY, M184V, T215S, K219EK	Y181C, H221Y	5.45	K65R, Y115F, M184V,T215S	Y181C, H221Y
ETH368	ZDV-3TC-NVP	5.98	—	G190A	—	LTFU	LTFU	LTFU	LTFU	LTFU	LTFU
ETH484	TDF-3TC-NVP	6.24	—	K103N	—	LTFU	LTFU	LTFU	LTFU	LTFU	LTFU
ETH216	ZDV-3TC-NVP	6.19	L210W K219Q	—	—	Dead	Dead	Dead	Dead	Dead	Dead
ETH465	ZDV-3TC-NVP	5.56	T215FIS	—	—	<2.18	VL <150 cp/ml	VL <150 cp/ml	LTFU	LTFU	LTFU
ETH144	TDF-3TC-EFV	5.84	T215S	—	—	<2.18	VL <150 cp/ml	VL <150 cp/ml	LTFU	LTFU	LTFU
ETH479	TDF-3TC-NVP	6.45	K219Q	—	—	<2.18	VL <150 cp/ml	VL <150 cp/ml	<2.18	VL <150 cp/ml.	VL <150 cp/ml
ETH218	TDF-3TC-EFV	5.03	K219Q	—	—	<2.18	VL <150 cp/ml	VL <150 cp/ml	LTFU	LTFU	LTFU
ETH354	TDF-3TC-EFV	5.35	T69D	—	—	<2.18	VL <150 cp/ml	VL <150 cp/ml	LTFU	LTFU	LTFU
ETH196	ZDV-3TC-EFV	5.39	—	—	N88D	<2.18	VL <150 cp/ml	VL <150 cp/ml	<2.18	VL <150 cp/ml	VL <150 cp/ml
ETH318	ZDV-3TC-NVP	5.99	—	—	F53L	<2.18	VL <150 cp/ml	VL <150 cp/ml	LTFU	LTFU	LTFU
ETH020	TDF-3TC-NVP	5.83	L210W	—	—	5.87	M184I, L210W	K103N, E138G, Y181C, G190A	LTFU	LTFU	LTFU
ETH205	d4T-3TC-NVP	6.36	T215FIS, K219Q	—	—	<2.18	VL <150 cp/ml	VL <150 cp/ml	<2.18	VL <150 cp/ml	VL <150 cp/ml
ETH406	d4T-3TC-EFV	5.45	—	K103N	—	<2.18	VL <150 cp/ml	VL <150 cp/ml	<2.18	VL <150 cp/ml	VL <150 cp/ml

^*^VL: log 10 copies (cp)/ml. Columns express drug resistance mutations DRM or no DRM detected (−) or reasons to no data. LTFU: lost-to-follow-up. TDF: tenofovir; zdv: zidovudine; EFV: efavirenz; NVP: nevirapine; 3TC: lamuvidine.

Fourteen out of the 18 patients with sDRM were still on ART at month 12, but six of them had no VL results. Patients with the RTI-sDRM had higher odds of virologic failure (defined as VL >150 copies/ml) after month six and 12 than those without RTI-sDRM (OR: 3.6; 95% CI: 1.2–11.1 and OR: 9.00; 95% CI: 1.9–43.3, the latter adjusted for tuberculosis co-infection). Also when the WHO treatment failure definition (VL >1000 copies/ml) was used, those with RTI-sDRM had higher odds of failure both at month six and 12 (OR: 6.5; 95% CI: 2.1–20.3 and OR: 7.4; 95% CI: 1.5–35.0, respectively). In the ITT analysis, patients with RTI-sDRM had significantly higher treatment failure rates at the six months, for both the 150 and 1000 copies/ml cut-off, than those without the mutations (OR: 2.9; 95% CI: 1.0–7.9 and OR: 3.8; 95% CI: 1.4–10.5, respectively), but not at month 12 (p = 0.053 and p = 0.099, respectively).

### Acquired DRM detected by PBSS

At month six, 47 sequences were obtained from 51 patients who had a VL >1000 copies/ml and attempted for sequencing. Of those, 37 (79%) had one to seven major NRTI and/or NNRTI DRMs, and no major PI DRM (NRTIs + NNRTI: 25 (68%); only NRTI: seven (19%); only NNRTI: five (14%)) (see Supplementary Table S1). At month 12, 25 (83%) of 30 sequences from 33 failing patients attempted for sequence analyses had one to eight major NRTI and/or NNRTI DRMs and no major PI DRM (NRTIs and NNRTI DRMs: 16 (64%); only NRTIs: 6 (24%); NNRTIs DRMs: three (12%)).

Among those patients with sDRM at baseline who reached month six and had VL data, six out of eight patients with NRTI sDRM and two of two with PI sDRM, but only one of the five patients with NNRTI sDRM (K103N) had undetectable viremia (Table 2).

### Baseline DRM detected by NGS

All NGS attempts were successful and baseline sequences were thus obtained from 109 patients. Patients with virological treatment failure (n = 71) had lower CD4+ T-cells at baseline than the virologic suppressors (n = 38) (112 cells/µl vs 150cells/µl; p = 0.02). No significant differences were found for other biological and demographic parameters in relation to the virologic treatment outcome.

Altogether NGS detected DRM at baseline in 28 patients (NRTI; n = 8; NRTI + NNRTI: n = 1; NRTI + INSTI: n = 1; NRTI + PI: n = 1; NNRTI: n = 10; PI: n = 3; INSTI DRM: n = 2; three drug classes: n = 2). NGS detected RTI or PI sDRM (>1% frequency) significantly more often (23.9%; 26/109) than PBSS (6.4%; 7/109) (p < 0.0001). In addition, INSTI DRM was found in five subjects.

The NGS DRMs (range one to three) were found in 23 (32.4%) of the 71 patients who at month six and/or 12 had a virological failure (>1000 copies/ml) (NRTI: n = 12; NNRTI: n = 9; PI: n = 5; INSTI: n = 4) (Table 3). Five (13.2%) out of 38 patients with undetectable viremia at month six and/or 12 had one DRM each at baseline (NRTI: n = 1; NNRTI: n = 2; PI: n = 1; INSTI: n = 1). There was no significant difference between patients with RTI and/or PI sDRM and those without the sDRM with regard to study sites, age, WHO clinical stage, CD4 cells or VL (data not shown). However, females had higher proportion of sDRM than males (19/60 vs 7/49, p < 0.05).

**Table 3:** Drug resistance mutations detected by next generation sequencing (NGS) or population based Sanger sequencing (PBSS) at baseline in 109 patients with virologic treatment failure (n = 71) or suppression (n = 38) at months six and/or 12.

PID	Gender	Age (years)	CD4 cells/ul	VL*	Outcome	NGS-DRM (%)**			PBSS-sDRM	
						PI	RTI	INSTI	NRTI	NNRTI
ETH066	Female	25	179	5.63	VF	G73C (1.0)	M184I (1.0)	Q148H (1.5)	—	—
ETH011	Male	45	128	4.20	VF	—	D67G (45.6), L74I (40.6), V75S (36.6)	—	—	—
ETH019	Female	28	22	6.40	VF	—	D67G (2.3), L74I (2.1), V75S (1.3)	—	—	—
ETH057	Female	24	143	6.36	VF	M46I (1.3)	D67G (1.0), L74I (1.1)	E138K (1.3)	—	—
ETH048	Female	37	83	5.97	VF	—	T215S (2.8)	E138K (2.1)	—	—
ETH010	Female	26	142	6.68	VF	I50L (1.4)	—	—	—	—
ETH064	Female	20	232	4.90	VF	—	Y181C (82.9), K219N (23.4)	—	—	Y181C
ETH043	Male	37	221	6.51	VF	L90M(8.7)	K65R (1.3)	—	—	—
ETH044	Male	38	192	5.77	VF	—	L74V (3.7), V75S (3.8)	—	—	—
ETH046	Male	36	6	6.70	VF	—	L74V (1.7), V75S (1.9)	—	—	—
ETH021	Female	20	174	6.93	VF	—	Y181C (38.3), Y188C (51.9)	—	—	Y181C, Y188C
ETH061	Male	15	90	4.28	VF	—	G190S (99.6)		-	G190S
ETH003	Female	26	219	4.75	VF	—	K101E (45.0)	—	—	—
ETH005	Female	25	175	4.65	VF	—	K101E (1.0)	—	—	—
ETH040	Male	37	21	5.83	VF	—	L210W (30.3)	—	L210W	—
ETH051	Female	27	234	5.02	VF	—	G190E (98.1)	—	—	—
ETH023	Female	49	76	ND	VF	—	T215I (2.2)	—	no data	no data
ETH035	Female	37	308	4.70	VF	—	K103N (3.7)	—	—	—
ETH026	Female	21	70	6.05	VF	—	T215S (99.3)	—	T215S	—
ETH052	Male	28	117	5.02	VF	—	Y181C (97.4)	—	—	Y181C
ETH062	Male	40	151	5.05	VF	—	—	Q148R (1.6)	—	—
ETH004	Female	21	146	4.06	VF	—	K101E (1.0)	—	—	—
ETH050	Female	30	8	5.71	VF	I54L (9.0)	—	—	—	—
ETH094	Female	40	115	5.38	VS	—	Y188C (1.2)	—	—	—
ETH099	Female	50	201	5.45	VS	—	K103N (24.0)	—	—	K103N
ETH077	Female	34	102	4.46	VS	—	Y115F (1.4)	—	—	—
ETH108	Female	28	62	6.58	VS	I50L (1.2)	—	—	—	—
ETH074	Female	25	21	5.38	VS	—	—	T66I (22.3)	no data	no data

*VL: viral load, (log10 copies/µl); VF: virologic failure (>1000 copies/ml); VS: virologic suppressor <2.18 log 10 copies/ml); **DRM% = drug resistance mutations and the proportion of mutant virus. DRMs were identified for NRTI, NNRTI and PI based on the 2009 WHO list for surveillance of TDR and for INSTI the Stanford drug resistance summary list; —: no DRM detected.

**Figure 2:**
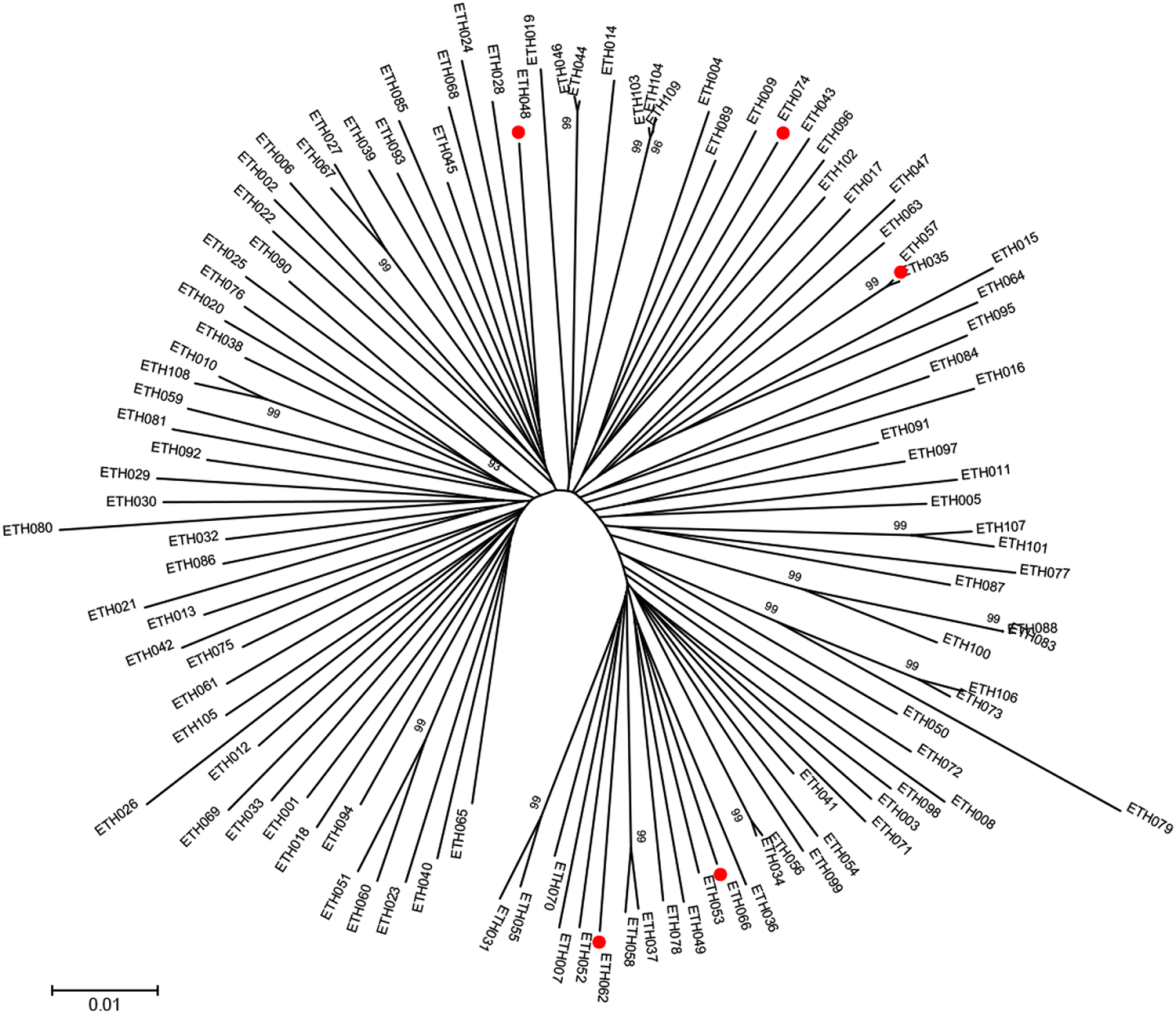
Maximum likelihood phylogenetic analysis of the 109 sequences generated by next generation sequencing. Sequences with INSTI mutations are highlighted in red for comparison with other sequences.

The INSTI-DRMs detected by NGS were E138K (n = 2; 1.3% and 2.1%, respectively), Q148R (n = 1; 1.6%), Q148H (n = 1; 1.5%), and T66I (n = 1; 22.3%) (Table 3). These patients were from all study sites, except Jimma and the Army unit (details of study sites depicted in the methodology section). No clustering was found among the viral strains with INSTI DRM (Fig. 2).

### Impact of baseline DRM detected by NGS and correlation with PBSS

From baseline samples of failing patients, NGS detected any RTI sDRM (at >1% frequency of the population) significantly more often (28.2%; 20/71) than PBSS (8.54%; 6/71) (p = 0.004; Fisher exact test) (Table 3). PBSS failed to detect six out of 14 (42.9%) sDRM from four patients despite that the NGS assay detected sDRM corresponding to greater than 20% of the viral population. These six sDRM were derived from four different patients. Patients who failed ART with >150 copies/ml at month six and/or 12 had more frequently one or more NRTI, NNRTI and/or PI sDRM by NGS at baseline as compared to the virologic suppressors (OR: 6.4; 95% CI: 1.6–26.4 adjusted for NRTI regimens and CD4 cell counts) (Table 3). This was also holds true when only patients with sDRM NRTI and/or NNRTI were considered (20/71 versus 3/38) (p < 0.05).

Next we checked whether sDRM detected by NGS appeared at virological treatment failure (>1000 copies/ml) at month six and/or 12, as determined by PBSS (Table 4). Among 16 patients who failed at month six, only six out of 25 NGS NRTI or NNRTI sDRM at baseline appeared at month six. All these sDRM were detected at a high proportion at baseline (T215S: 99.3%, L210W: 30.3%, Y181C: 38.3%, Y181C: 97.4%, G190S: 99.6%, Y181C: 82.9%). However, other six sDRM at high baseline proportions were not detected at month six or 12 (K101E: 45.0%, D67G: 45.6%, L74I: 40.6%, G190E: 98.1%; K219N: 23.4%, Y188C: 51.9%).

**Table 4:** Surveillance drug resistance mutations associated with reverse transcriptase inhibitors identified by next generation sequencing at baseline and direct Sanger sequencing at treatment failure month six and 12.

PID	First-line regimen	Minor DRM at baseline		Month 6			Month 12		
		NRTI	NNRTI	VL*	NRTI	NNRTI	VL*	NRTI	NNRTI
ETH003	TDF-3TC-EFV	—	K101E (45.0)	<2.18	VL <150 cp/ml	VL <150 cp/ml	4.7	—	V106M
ETH004	ZDV-3TC-NVP	—	K101E (1.0)	3.86	L210LW	P236LP	2.32	VL <1000 cp/ml	VL <1000 cp/ml
ETH005	ZDV-3TC-NVP	—	K101E (1.0)	< 2.18	VL <150 cp/ml	VL <150 cp/ml	4.57	—	K103KN, V179T, G190GA
ETH011	d4T-3TC-NVP	D67G (45.6), L74I (40.6)	—	<2.18	VL <150 cp/ml	VL <150 cp/ml	4.39	K219Q	E138A
ETH019	TDF-3TC-EFV	D67G (2.3), L74I (2.1)	—	6.4	M184I	L100I, K103N	5.92	K65R, M184I	L100I, K103N, M230L
ETH021	TDF-3TC-EFV	—	Y181C (38.3), Y188C (51.9)	6.38	K65R, K70KE, M184IV	K103N, Y181C, G190S, F227L	6.8	No sample	No sample
ETH023	ZDV-3TC-NVP	T215I (2.2)	—	<2.18	VL <150 cp/ml	VL <150 cp/ml	3.09	L210W	—
ETH026	TDF-3TC-NVP	T215S (99.3)	—	5.15	Y115YF, M184V, K219KE,T215S	Y181C, H221Y	5.45	K65R, Y115F, M184V, T215S,	Y181C, H221Y
ETH035	TDF-3TC-EFV	—	K103N (3.7)	<2.18	VL <150 cp/ml	VL <150 cp/ml	<2.18	VL <150 cp/ml	VL <150 cp/ml
ETH040	TDF-3TC-NVP	L210W (30.3)	—	5.87	M184I, L210W	K103N, E138G,Y181C, G190A	No sample	No sample	No sample
ETH043	TDF-3TC-EFV	K65R (1.3)	—	5.75	—	K103N, V106M	No sample	No sample	No sample
ETH044	ZDV-3TCff EFV	L74V (3.7)	—	6.24	—	K103N	LTFU	LTFU	LTFU
ETH046	TDF-3TC-EFV	L74V (1.7)	—	5.66	M184V	K101KE, V106M, G190A	Dead	Dead	Dead
ETH048	TDF-3TC-NVP	T215S (2.8)	—	5.01	A62V, K65R, M184V	K103N, Y181C	Dead	Dead	Dead
ETH050	d4T-3TC-NVP	—	—	5.14	K219Q	—	<2.18	VL <150 cp/ml	VL <150 cp/ml
ETH051	ZDV-3TC-NVP	—	G190E (98.1)	4.93	K219Q	—	Not done	Not done	Not done
ETH052	ZDV-3TC-NVP	—	Y181C (97.4)	4.55	K70KR, M184V	A98G, Y181C, G190A	LTFU	LTFU	LTFU
ETH057	ZDV-3TC-NVP	D67G (1.0), L74I (1.1)	—	2.3	VL <1000 cp/ml	VL <1000 cp/ml	<2.18	VL <1000 cp/ml	VL <1000 cp/ml
ETH061	ZDV-3TC-NVP	—	G190S (99.6)	5.32	—	G190S	5.29	—	—
ETH062	TDF-3TC-EFV	—	—	2.25	VL <1000 cp/ml	VL <1000 cp/ml	3.4	K65R	V106M, V179D
ETH064	ZDV-3TC-NVP	K219N (23.4)	Y181C (82.9)	3.7	M184V	V108I, Y181C	2.36	VL <1000 cp/ml	VL <1000 cp/ml
ETH066	ZDV-3TC-NVP	M184I (1.0)	—	2.35	VL <1000 cp/ml	VL <1000 cp/ml	No sample	No sample	No sample

^*^VL: log 10 copies (cp)/ml. Columns at month six and 12 express DRM or no DRM detected (−) or reasons to no data; TDF: tenofovir; ZDV: zidovudine; EFV: efavirenz; NVP: nevirapine; 3TC: lamuvidine; LTFU: lost-to-follow-up.

## Discussion

The present study is the first countrywide representative survey of transmitted drug resistance (TDR), based on the first large national ART cohort study in Ethiopia^13,14^. Analysing 461 *pol* sequences by PBSS, we found a low frequency, 3.9%, of treatment-naïve patients with sDRM. In a selected sub-set of 109 patients, additional DRMs were found by NGS, including major INSTI DRMs in five patients. Patients with TDR failed therapy more frequently both in OT and ITT analysis, suggesting a clinical impact of these mutations.

By PBSS, NRTI and NNRTI sDRM were found as expected, but also non-polymorphic accessory PI sDRM in two patients, despite the infrequent use of PI in Ethiopia in 2009–2011. An inclusion criterion in the ACM cohort was self-reported no earlier use of ART. If correctly self-reported, the prevalence of TDR was 3.9% and no regional difference within Ethiopia was observed. However, it shall be emphasized that the patients were recruited in 2009–2011 and that the present situation of pretreatment resistance (PDR) may have been changed. TDR in LMIC has increased, primarily NNRTI TDR, over time in sub-Saharan Africa (SSA)^9^. In addition, it should be noted that our patients had low CD4 cell counts at start of ART and were most likely not newly infected. Therefore, the TDR rate might be underestimated in our study since some drug resistant variants frequently disappear from the major viral population after a period of no ART. The increase has been steepest in east Africa up to a 7.4% prevalence eight to nine years after rollout of ART. An update until 2016, but now including all PDR, confirms this trend and the predictions of the prevalence of NNRTI PDR for 2016 were 11% (95% CI 7.5–15.9) and 15.5% (95% CI 7.7–28.8) in Southern- and Eastern-Africa, respectively^9^. Data from Ethiopia was however not included in these reports. Smaller and regional studies using PBSS have reported low frequencies, 3.3% in 2003^11^ and 0% in 2005^15^, which increased in later studies, 5.6% in 2008^12^, and 7.2% in 2010^16^. Our nationwide data from 2009–2011 in a larger number of patients did not however suggest an increasing trend of TDR in Ethiopia up to then.

A higher number of sDRM was identified by NGS, which is in line with our earlier report of a high detection rate (6.5%) of NNRTI TDR in Addis Ababa, 2009–2010, using a sensitive allele-specific PCR^10^. Thus, additional DRMs were detected in 17 patients selected for the NGS assay. Of these, mutated viral populations representing more than 20% were found in four patients represented, which should have been possible to be able to detect with our PBSS assay. Although the selection of these patients were biased, the discrepancy between PBSS and NGS suggests that NGS facilitates detection of HIV-1 sDRMs in LMICs and reveals a higher prevalence of PDR to the same or lower cost if high-throughput approaches are used^17^.

In a study conducted on small number of patients (n = 45) from Gondar, Ethiopia recruited in 2008, using PBSS, no major INSTI DRM was found^18^. Interestingly, in our study major INSTIs mutations (T66I, E138K, Q148R, and Q148H) were found in five patients albeit at a low abundance. At the time when the study started (2009–2011), to our knowledge, no patient in Ethiopia had been treated with an INSTI and still these drugs are not an integrated part of the Ethiopian ART regimes. It cannot be excluded that INSTIs DRMs have been introduced in Ethiopia through patients who have been treated outside the country. However, our phylogenetic analysis showed no clustering of the strains with INSTIs DRM and the patients came from five different study sites all over Ethiopia. It seems therefore unlikely these strains have been transmitted from INSTI treated subjects. Also, we found no evidence of cross-contamination of INSTI-resistant strains in our laboratory, which is strictly separated from the clinical diagnostic laboratory. A possibility is that wild-type HIV-1C strains in Ethiopia may harbor low abundance of INSTI DRMs. All of the identified DRMs alone or in combination associated with resistance to raltegravir and/or elvitegravir.

Recently, dolutegravir (DTG) has been planned to be used in some African countries, as fixed dose combination given once daily. Importantly, the INSTI DRM E138K contributes to reduced susceptibility to DTG in combination with other INSTI DRM. Also, Q148R and Q148H are associated with low-level or intermediate resistance to DTG, which should be administered twice daily if these DRM are present. A similar pattern is found for cabotegravir and bictegravir^19,20^. Our finding warrants therefore expanded analysis of minor quasispecies with regard to INSTI DRMs in different African patient populations in order to identify how often such low abundance DRM can be find.

The impact of preexisting INSTI DRMs on clinical treatment response has been discussed earlier^21,22^. E.g. the E157Q mutation has been reported in 1.7% and 5.6% of viral sequences from ART-naïve patients, depending on subtype^23^ and been implied to affect treatment response^24^. On the other hand, low abundance INSTI DRMs were not shown to have impact on treatment outcome^21,22^. However, these latter studies used allele-specific PCR detecting a significantly lower proportion of mutated virus than our NGS method, which has 1% cut-off. Therefore, a potential clinical impact of our findings still remains to be evaluated.

Patients with baseline *pol*-sDRM failed ART more frequently at month six. Also additional sDRM were identified pre-ART with the NGS assay. A dose-effect association between the level of low-abundance NNRTI-resistant mutants and a 5% threshold of mutant frequency has been suggested to be clinically relevant^25^. In our study, the number of patients with NGS sDRM was too small to allow identification of a threshold. However, among 16 patients who failed at month six, only sex out of 25 NGS NRTI or NNRTI sDRM at baseline were detected at month six by PBSS. All these six sDRM had been detected at a high proportion at baseline. The lack of detection of the minor sDRM in the follow-up samples could possibly indicate they had a limited or no impact on the emergence of drug resistance at the follow-up time points. However, further study is recommended to assess their impact as a secondary mutation for the emergence of DRM.

In conclusion, we have analysed TDR in the largest nationwide Ethiopian cohort so far and found that in 2009-2011 the rate was still low, 3.9%, using PBSS, but TDR before treatment was associated with a poorer treatment outcome. Also, our NGS results showed that the rate of 3.9% is an underestimation although we could not confirm that the low abundance DRM had a clinical impact. Interestingly, we identified preexisting INSTI DRM in wild-type HIV-1C from treatment naïve patients. Our data shows the importance of surveillance for TDR and PDR in LMIC and suggests an added value of using high-throughput NGS in such studies.

## Material and Methods

### Study Population

**Figure 3:**
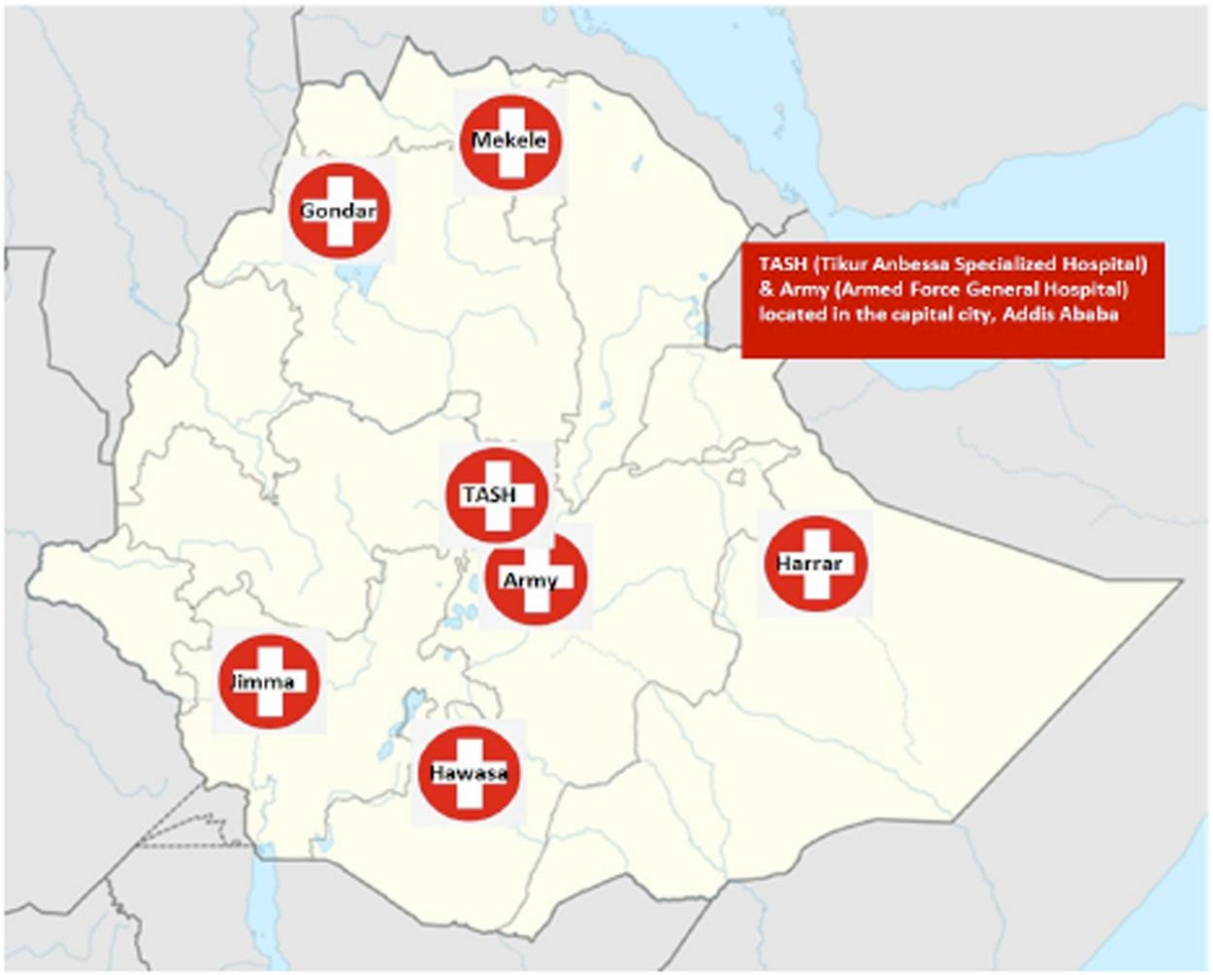
Geographical locations of seven university affiliated hospital study sites in Ethiopia.

Through October 2009 to December 2011, a total of 874 ART naïve patients were recruited to the Advanced Clinical Monitoring (ACM) of ART in Ethiopia cohort, and started ART, as per the national guideline^4^. The subjects were from seven universities^13,14^, distributed geographically all over the country: Tikur Anbessa Specialized Hospital in Addis Ababa- Central region; Gondar– Northwest; Jimma– West; Mekelle– North; Harrar– East; Hawassa– South; the Army unit providing service to mobile military staff, which is located in Addis Ababa (Fig. 3). Our study was conducted on 490 subjects (age ≥ 14 years), randomly selected after stratifying by study sites (70 from each site), who were followed until the end of 2013 (Table 1). The following FDCs were given: TDF + 3TC + EFV (n = 222), TDF + 3TC + NVP (n = 39), ZDV + 3TC + EFV (n = 60), ZDV + 3TC + NVP (n = 144), stavudine (d4T) + 3TC + EFV (n = 15), d4T + 3TC + EFV (n = 9), and abacavir (ABC) + 3TC + EFV (n = 1).

Clinical and routine laboratory tests were performed at the study sites. Ten ml whole blood was collected and processed for each patient at baseline, month six and 12. Plasma samples were centrally stored at the Ethiopian Health and Nutrition Research Institute (EHNRI) at −80 °C after transport on dry ice. Quantification of VL was performed by NucliSENS easyQ^®^ HIV-1 Nucleic Acid Sequence-Based-Amplification (NASBA) (BioMérieux Diagnostics) with a detection limit of 150 HIV-1 RNA copies/ml. CD4 T-cell count was determined at the hospital laboratories by BD FACSCalibur machines (Becton Dickinson, San Jose, USA). Data was entered into a site database and later uploaded to the central database at EHNRI, from which the following data were extracted: sex, age, WHO clinical stage, ART regimen, CD4 cell count and VL.

### Population-based Sanger sequencing (PBSS)

PBSS was attempted on 490 baseline samples as well as on 51 and 33 samples with VL ≥1000 copies/ml at month six and 12, respectively. HIV RNA was extracted from 140 µl plasma using the QIAamp^®^ RNA extraction mini-kit (Qiagen, Hilden, Germany). cDNA synthesis was done using RevertAid H-minus reagents (Life technologies, Paisley, UK). The first-round PCR was done using JA203F-C (forward) and JA206R-C (reverse) primer pair, followed by the second-round PCR, using JA204F-C (forward) and JA205R-C (reverse) primer pair^26^. The amplified fragments were purified (QIAquick PCR Purification Kit, Qiagen, Hilden, Germany) and sequenced with JA204F-C and JA205R-C PCR-primers plus PR2R (5′-GGATTTTCAGGCCCAATTTTTG-3′) and RT07 (5′-AAGCCAGGAATGGATGGCCCA-3′). This method has been used extensively at our laboratory. Positive PCR reactions are obtained in 100% of plasma samples containing the equivalent of 500 HIV-1 RNA copies per PCR reaction. In practice the assay gives positive results in the vast majority of plasma samples containing >500 copies/ml with a sensitivity to detect 20% mutated variants in the viral population. A comparison between the original assay^26^ and our slightly assay modified with primers specifically designed for HIV-1C has shown equal results.

Sequences were aligned, edited and analysed using the BioEdit software version 7.2.6.1 (http://www.mbio.ncsu.edu/bioedit/bioedit.html). Primary DRM were identified by the calibrated population resistance tool (http://cpr.stanford.edu/cpr.cgi) at Stanford HIVDR Database. Acquired DRM were identified by the Stanford HIVdb Program (hivdb.stanford.edu). DRMs associated with NRTI-, NNRTI-, and PI- drug classes were considered in this assay.

### Next generation sequencing (NGS)

NGS was performed on 109 baseline samples of all patients, who had viremia at month six and/or 12 (n = 71), and from randomly selected patients with undetectable viremia (n = 38), as described^27^. In brief, fragment I (HXB2: 790 – 5096) covering *Gag-pol* was amplified, gel purified, and fragmented on the Coveris S200 followed by library preparation using NEBNext UltraTM DNA library Prep Kit. Forty-eight libraries were then pooled at equimolar (10 nM each) and run on Illumina HiSeq. 2500. The FASTQ file was demultiplexed and the consensus sequence was created for each sample followed by realignment again with the consensus sequence as input. The variant calling was performed at amino acid (AA) level. Only AA covering 5000× per position was considered quality passed. Based on the error calculation generated by PCR and NGS, any mutation >1% was considered. WHO list of DRM for surveillance of TDR was used to interpret sDRM for NRTIs, NNRTIs, PIs, and the Stanford drug resistance summaries for INSTIs (hivdb.stanford.edu).

### HIV-1 subtyping and phylogenetic analysis

Subtyping was done by Recombinant Identification Program (http://www.hiv.lanl.gov/content/sequence/RIP/RIP.html), REGA HIV Subtyping Tool v3, (http://dbpartners.stanford.edu:8080/RegaSubtyping/stanford-hiv/typingtool) and COMET HIV-1 (http://comet.retrovirology.lu). Maximum likelihood phylogenetic analysis was performed using Molecular Evolutionary Generics Analysis version 7.0 (MEGA 7) software.

### Treatment outcome measures

The outcomes at month six and 12 were analysed by both on-treatment (OT) and intention-to-treat (ITT) approaches. In the OT analysis, two VL cut offs were used for the definition of virological treatment failure; >150 copies/ml and >1000 copies/ml, respectively. For ITT, treatment failure was defined as either failure to attain undetectable viremia (either <150 copies/ml or <1000 copies/ml), LTFU or death.

### Statistical analysis

Descriptive statistics (mean, median, standard deviation, percentiles for numerical variables, frequencies and percentages for categorical variables) were used to summarize sociodemographic, clinical, immunological, and virological parameters. Prevalence and types of DRM at baseline were investigated for their possible relationship with sociodemographic and clinical characteristics using t-test (for continuous variables), and Chi-square or Fisher’s exact test (for categorical variables). The impact of pretreatment sDRM (RTI, PI) detected by PBSS and NGS assays on virologic treatment outcome at month six and 12 was assessed by using a multivariable model testing for different confounding factors including gender, age, WHO clinical stage, functional status, TB, CD4 cell count, baseline VL, and NRTI regimens. P-value < 0.05 was considered statistically significant. Data analysis was performed using STATA software 14 (Stata Corp. College Station, Texas, USA).

### Ethical approval and informed consent

Scientific and ethical approvals were obtained from the National Research Ethics Review Committee in Ethiopia (3.10|528|06) and the Institutional Review Board (IRB) of EHNRI (Reference No. E.H.N.R.I 6.13/163). Written informed consent was obtained from all patients. All the methods were performed in accordance with approved institutional guidelines.

The sequences generated by Sanger sequencing in this study are deposited in Gene Bank [accession numbers: MG009597-MG010057].

## Electronic supplementary material

Supplementary Table S1
