# Supplementary material for: Pretreatment drug resistance in a large countrywide Ethiopian HIV-1C cohort: a comparison of Sanger and high-throughput sequencing
Source: Sci Rep. 2018 May 15;8:7556. doi: 10.1038/s41598-018-25888-6 (PMC5954158; doi:10.1038/s41598-018-25888-6)
Supplement: Supplementary file 1 — Supplementary Table S1 [file 41598_2018_25888_MOESM1_ESM.doc]

**Pretreatment drug resistance in a large countrywide Ethiopian HIV-1C cohort: a comparison of Sanger and high-throughput sequencing**

**Authors:** Nigus Fikrie Telele^1,2*^, Amare Worku Kalu^1,2^, Solomon Gebre-Selassie^2^, Daniel Fekade^3^, Samir Abdurahman^4^, Gaetano Marrone^5^, Ujjwal Neogi^1^, Belete Tegbaru^6^, Anders Sönnerborg^1,5^.

**Supplementary Table S1: Frequency of NRTIs- and NNRTIs-associated drug-resistance mutations (DRM) observed in treatment failure participants at months six months and 12 after initiation of antiretroviral therapy.**

| NRTI-associated DRMs | | | | | | | | | | | | | | | | | | | | | | | | | | | | |
| --- | --- | --- | --- | --- | --- | --- | --- | --- | --- | --- | --- | --- | --- | --- | --- | --- | --- | --- | --- | --- | --- | --- | --- | --- | --- | --- | --- | --- |
|  | K219Q/ KQ/N/EK | | M184V/I | | K65R/N | | T215F | | T215S/I | | | T69N/I | | Y115F | | F116Y | | K70R/E/  KR/EK/KT | | V75I | | | D67G /DN | | A62V /AV | | M41L | |
| Month 6 | 8 | | 14 | | 10 | | - | | 1 | | | 1 | | 3 | | 1 | | 4 | | 2 | | |  | | 1 | | - | |
| Month 12 | 5 | | 9 | | 8 | | - | | 3 | | | - | | 2 | | - | | 2 | | 2 | | | 2 | | 3 | | - | |
| NNRTI-associated DRMs | | | | | | | | | | | | | | | | | | | | | | | | | | | | |
|  | K103N | Y181C | | V106M | | G190A/E /I/S/AG | | V108I /IV | | K101E | V179D  /E/T | | V90I | | Y188C /NY | | A98G /AG | | E138A /G | | L100I | H221Y | | P225H | | M230L | | K238T |
| Month 6 | 15 | 11 | | 8 | | 11 | | 3 | | 3 | 4 | | 2 | | 2 | | 1 | | 2 | | 1 | 1 | | 2 | | 1 | | 1 |
| Month 12 | 6 | 7 | | 6 | | 7 | | - | | 2 | 4 | | - | | 2 | | 1 | | 1 | | 1 | 3 | | - | | 1 | | 2 |
